# Powerful Leverages and Counter-Currents in the Unborn Child Spiritual Care: A Qualitative Study

**DOI:** 10.5539/gjhs.v7n1p122

**Published:** 2014-08-22

**Authors:** Tooba Heidari, Saeideh Ziaei, Fazlollah Ahmadi, Eesa Mohammadi

**Affiliations:** 1 Department of Midwifery & Reproductive Health, Faculty of Medical Sciences, Tarbiat Modares University, Tehran, Iran; 2 Department of Nursing, Faculty of Medical Sciences, Tarbiat Modares University, Tehran, Iran

**Keywords:** prenatal care, unborn child, spirituality, facilitators, barriers

## Abstract

In different cultures, pregnancy, birth and motherhood are perceived as spiritual events through their miraculous processes and create an ideal context for spiritual enrichment. However, studies on spirituality and birth are at very early stages. The purpose of this study was to understand the facilitators and barriers of the unborn child spiritual care in Iranian women. Twenty-two mothers with live pregnancy experience who were willing and able to share their life stories were selected purposefully in Tehran (Iran) from May 2012 to April 2013. Qualitative content analysis was used to analyze 27 interviews. Active and passive acquisition of information, inner inspirational messages, receiving effective support from the people around as well as modeling of self and significant others created “powerful leverages” to accelerate mother for caring her unborn child. “Counter-currents” in the form of unsuitable physical conditions during pregnancy, poor economic and social conditions, unsuitable psychological and cognitive conditions and finally understanding unsuitable ideological conditions of the self and care giver were identified as barriers. Iranian cultural and religious perspective on the unborn child physical and mental influence from mother has an important role in mother’s self-care behaviors during pregnancy. It seems that using interdisciplinary professionals’ skills based on understanding facilitators and barriers of mother care of the unborn child can lead to providing comprehensive prenatal care according to mothers’ cultural, religious and social context.

## 1. Introduction

The majority of recent studies on spiritual health have focused on chronic diseases and those with end-stage diseases, while in different cultures, pregnancy, birth and motherhood are perceived as spiritual events through their miraculous processes and create an ideal context for spiritual enrichment (Callister & Khalaf, 2010; Moloney, 2007; Baumiller, 2002; Hall & Taylor, 2004). However, studies on spirituality and birth are at very early stages (Linhares, 2005). Most of the existing studies have referred to religion and spirituality as a source of controlling high-risk behaviors and coping with adverse psychological conditions during pregnancy (Teman et al., 2011; Elizabeth Jesse et al., 2006; Jesse & Swanson, 2007; Mann et al., 2007a, 2007b, 2008; Cowchock et al., 2011). Very few studies that have focused on the stories of mothers’ lives during pregnancy show that they believe there is a spiritual nature for their unborn child (Hall, 2006) and this will result in some changes in religious and spiritual behaviors during pregnancy (Callister & Khalaf, 2010). Receiving the spiritual experiences of mothers from different cultures during pregnancy and birth is an important solution for improving the care of their unborn child. Health care providers need to provide better care for them by relying on pregnant mothers ‘religious and spiritual beliefs (Callister & Khalaf, 2010; Callister, 2004). However, the important point here is that the midwives and nurses are expected to have a holistic view on the care provided by them and this will be achieved if the unborn child and the care of its spirit are also considered (Hall, 2010); in other words, the care before conception and during prenatal period is the first preventive care that human receives in that providing such a care is a touch of the future (Dooley & Ringler Jr, 2012). One of the main steps in providing comprehensive care during pregnancy is to understand the factors that affect mother’s caring for her spirit as she is the first and most important person who feels the concern of unborn child protection and the need to meet comprehensive needs of him/her (Darvill et al., 2010). Review of the literature revealed that no direct study, either qualitative or quantitative, has been conducted in this regard.

Studies suggest that the best way to understand the needs of women and provide professional health services for them is to listen to their inner experiences (Brems & Griffiths, 1993). To provide successful prenatal care, mothers’ underlying beliefs and cultural-social context should be considered (Finlayson & Downe, 2013). We attempted to understand the facilitators and barriers of the unborn child spiritual care in Iranian mothers. Understanding women’s perspectives on prenatal care can help designing and developing necessary policies to reduce barriers and achieve comprehensive care for the mother and her child (Phillippi, 2009; Gollenberg et al., 2008).

## 2. Methods

In this study, to understand the factors affecting the unborn child spiritual care by pregnant mothers, the qualitative research method was used as this method provides tools for examining these factors. This method facilitates entering people’s real life, contemplating on what people say and encourages participants to freely describe their feelings, experiences and performances. Less known issues based on cultural and religious context such as spiritual health in mother and unborn child are appropriate areas for qualitative studies (Corbin & Strauss, 2008; Kairuz & Crump, 2007).

Twenty-two mothers with live pregnancy experience (They were pregnant or had experienced pregnancy) who were willing and able to share their life stories were selected purposefully in Tehran (Iran) from May 2012 to April 2013. Therefore, mothers who were the source of rich experience on pregnancy and could provide a better understanding of life and social interactions were selected (Krippendorff, 2004).

Data were extracted from in-depth unstructured interviews, and interview questions changed according to the participants’ answers. Interviews conducted in a friendly environment, away from any threat at the women’s own homes or in a private room at the healthcare centre or in a place that mothers selected. The first interview question was: “Please tell me about the story of your life during pregnancy”. This question was open-ended to make the participants feel comfortable and encouraged to express their inner experiences of pregnancy freely. In the follow-up questions, gradually more specific topics were asked. During the interview, exploratory questions such as “Can you give me a real experience so that I can understand what you mean better?” Or “What do you mean?” were used so that data could be explored deeper and richer. Interviews were recorded with the knowledge and consent of participants. If data needed clarification, a second interview was performed with the participant. Interviews lasted 15 to 90 minutes. Sampling continued until data saturation, where no other information or new categories were identified (Holloway & Wheeler, 2010).

Medical Research Ethics Committee of the Tarbiat Modares University (Iran) issued the permission to conduct this study. Informed written consent was obtained from all participants. Before each interview, participants were clearly briefed on the importance of the study and its objectives. All interviews were recorded after informing the participants and obtaining permission. The participants were assured of the confidentiality of their information and that they could withdraw from the study at any time. If participants were eager, the way to achieve results was explained to, as well.

At the time of data collection, data analysis was conducted by conventional content analysis method (Krippendorff, 2004). Thus, after the end of each interview, the audio file was immediately transcribed verbatim along with verbal references such as laughing, crying, sighing and silence in MS-Word environment and imported to OneNote 2007. Data analysis began by reading interview texts several times so the researcher immersed in data. Then data were broken up into meaning units. After repeated reviewing of meaning units, the appropriate codes for each unit were written. The researcher also wrote analytic memos to contemplate on the data and to direct the research process. Data were classified based on thematic similarities and differences of primary codes and they were compressed as much as possible. This process continued throughout all analysis stages so that main categories that were more conceptual and more general were achieved. Finally themes were abstracted.

Study stages and process were registered and reported step-by-step as precisely as possible for confirmability and dependability of data, and step-by-step control of the research process was performed by two professors who were expert in qualitative research method. Prolonged engagement with the research subject, member check and external check led to increased data credibility. Using sampling technique with the highest diversity in terms of age, gestational age, gravidity, the number of children, elapsed time of delivery, occupation and education level helped the transferability of findings.

## 3. Results

**Figure 1:**
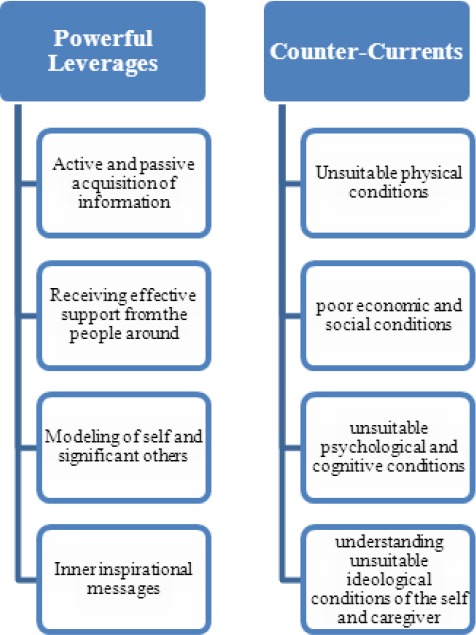
The facilitators and barriers of the unborn child spiritual care in Iranian women

The findings are based on information obtained from 27 interviews with 22 participants. Participants’ characteristics are shown in Table 1. The research results showed that concepts such as active and passive acquisition of information, receiving effective support from the people around, modeling of self and significant others are important and inner inspirational messages created “powerful leverages” to accelerate mother for caring her unborn child. “Counter-currents” in the form of unsuitable physical conditions, poor economic and social conditions, unsuitable psychological and cognitive conditions and ultimately understanding unsuitable ideological conditions of the self and caregiver were identified as barriers (Figure 1).

**Table 1:** Participants’ characteristics

Variable	Characteristics
The number of individual interviews	22 (9 pregnant mothers, 13 mothers delivered)
Age	24 to 47 years old
Gestational age	First, second and third quarter
The number of pregnancies	1 to 4
Elapsed time of delivery	6 months to 22 years
Education Level	From Diploma to PhD
Field of Study	Humanities, Medical Sciences and Technology
Occupation	Housewife, employees, student

### 3.1 Powerful Leverages (Bridges)

#### 3.1.1 Active and Passive Acquisition of Information

Acquisition of information was a remarkable experience that was clear in the statements of all mothers. Such an experience has three dimensions explained as follows:

3.1.1.1 The Effect of Searching Information and Self-Learning on the Formation of Spiritual Beliefs and Behaviors

Mothers tried to obtain the information required about the factors affecting unborn child spiritual development from available resources so that they could provide better care for their child:

*“During my pregnancy, I usually tried to read books containing specific recommendations such as reading the Quran, eating certain things or performing rituals at certain times for having good effect on the baby’s spirit”* (Participant 11, 30 years old, history of one pregnancy).

The Internet was also an interesting and appropriate source for mothers who had the ability to use it so that they could obtain updated information about the intrauterine world:

*“I always read articles on the Internet to know which parts of the body develop in each week of pregnancy, how much he understands in each week, how I should behave to be better for him/her. I did everything possible for him/her”* (Participant 2, 28 years old, history of one pregnancy).

3.1.1.2 The Effect of Transferred Information From Media and Society on the Formation of Spiritual Beliefs and Behaviors

Some mothers acquired information passively from the environment:

*“Some people in pregnancy exercise classes said if someone read Surah Al Asr during her pregnancy, her baby would be patient; I did so when I was pregnant with my son, it was very effective”* (Participant 20, 31 years old, history of one pregnancy).

The media had an important role in providing information required by mothers about unborn child spiritual development:

*“On a TV series, there was a couple that before making a baby went to a garden, took some privacy, said prayers and recited the Quran to make their spirit ready for pregnancy, I was very impressed”* (Participant 3, 29 years old, first pregnancy, 30 weeks).

3.1.1.3 The Effect of Religious Teachings on the Formation of Spiritual Beliefs and Behaviors

Iranian mothers’ religious context led them to adopt particular spiritual behaviors:

*“Halaal (religiously allowed /lawful) income is important for me. It is believed in our religion that haraam (religiously forbidden and disallowed) food may negatively affect the baby”* (Participant 17, 33 years old, second pregnancy, 10 weeks).

#### 3.1.2 Receiving Effective Support from the People Around

Supporting by significant others constitutes a great part of daily experiences of mothers:

3.1.2.1 The Role of Family as Company and Support

Family, especially the spouse, as the primary supportive source help mother in unborn child spiritual care:

*“While I was pregnant, especially in the last three months when I was really heavy and my conditions were intolerable, my husband was really very helpful. Most of the time he took me out for a ride so I don’t feel lonely at home. He tried to ease my discomfort as much as possible and not to upset me. Certainly discomfort is transmitted to the baby”* (Participant 12, 28 years old, history of one pregnancy).

3.1.2.2 The Role of Friends and Relatives as Company and Support

The people around encouraged and supported mother in providing better care of the unborn child:

*“During the whole time of my pregnancy, one of my friends spent so much time with me. She always searched the Internet, calling me saying, for example, ‘eat candies; it makes the baby feel happy when he/she hears the crunching sound of eating candy from inside the womb’…”* (Participant 2, 28 years old, history of one pregnancy).

#### 3.1.3 Modeling of Self and Significant Others

Influenceability from self and significant others were some of the major experiences of the mothers that expedite the movement of the mothers in spiritual care of unborn child:

3.1.3.1 The Effect of Personal Experiences as a Model on the Formation of Spiritual Beliefs and Behaviors

Observing the effect of spiritual considerations on unborn child resulted in developing a suitable model for mother to create spiritual beliefs and behaviors:

*“My first son, only 6 years old, stands next to me and prays, this means he has seen and felt me in the foetal period. The child sees and understands the character and temperament of his/her mother”* (Participant 5, 39 years old, second pregnancy, 27 weeks).

3.1.3.2 The Effect of Family and People around as a Model on the Formation of Spiritual Beliefs and Behaviors

The clear patterns in the family or people around suggesting the unborn child influence from mother’s behaviors during pregnancy increase mother’s motivation to use effective behaviors on the child spirit:

*“A relative of mine who reads her regular prayers began to read the Quran and pray more when she found out she was pregnant. She gave birth to two children and her children are really calm. Because of this I think if I do so, my baby will become also calm”* (Participant 15, 28 years old, first pregnancy, 6 weeks).

3.1.3.3 The Effect of Society as a Model on the Formation of Spiritual Beliefs and Behaviors

Some social problems such as sin and moral deviations were stressful for parents and made them double their efforts to institutionalize good traits in the child during pregnancy:

*“I think the reason that some girls and boys appear in the community with unsuitable appearance (provocative) is that their parents did not have strong beliefs. I learned from my mother to pray and fast. Well, these are very important. They make me teach my kid to behave like me and his father. This is what we can do”* (Participant 5, 39 years old, second pregnancy, 27 weeks).

#### 3.1.4 Inner Inspirational Messages

Inspiring internal reflections were commonly observed in mothers’ live experiences:

3.1.4.1 Understanding the Unborn Child Presence and Monitoring

Mothers said about an understanding that implied the unborn child influence in their actions, which is reflected in two main themes:

3.1.4.1.1 Feeling Mother’S Behavioral Monitoring by the Unborn Child

Mothers from the time of conception and early weeks of pregnancy felt that their behaviors were monitored by the child:

*“During my pregnancy, I noticed any behavior very carefully. I felt my baby is looking at me. I thought anything affected him/her”* (Participant 22, 43 years old, history of one pregnancy).

3.1.4.1.2 Understanding the Effect of Active Presence and Viability of the Unborn Child on the Mother’s Actions

Mothers believed that experiencing, feeling and understanding the reactions of the child in the form of his/her special movements during or following calling his name or other acts suggested the unborn child living presence, awareness, understanding and reacting to them:

*“When I was talking with my child, when I called his/her name, he kicked; I saw his reaction on my tummy (with emphasis), but some people didn’t believe me until I showed them the reaction of my baby…”* (Participant 21, 47 years old, history of one pregnancy).

3.1.4.2 Responsibility

Mothers feel responsibility for the care of their unborn child soul. This inner constructive feeling was expressed in the form of two concepts:

3.1.4.2.1 Mother’s Anxiety due to the Concern of the Unborn Child Being Influenced by Her Behavior

In case of negligence in spiritual behaviors, mothers got upset and had a heavy conscience for not properly doing their motherly responsibility and they were filled with fear. This severe conscientious control made mothers more sensitive to avoid negative behaviors:

*“Sometimes Satan deceives human, some thoughts occur to mind such as to be upset of others’ success. In such cases I quickly say: Oh, God please forgive me. I don’t know. It seems to me that it would instantly affect the child, therefore I have so much heavy conscience, lest my thought affects the child”* (Participant 16, 25 years old, first pregnancy, 8 weeks).

3.1.4.2.2 Responsibility and Commitment to the Child’s Future

The concern for creating a desirable future for the child resulted in raising commitment in mother:

*“I feel I don’t belong to myself anymore, all my concern is to devote what I’ve learned to him/her to create a better world for him/her”* (Participant 19, 37 years old, third pregnancy, 5 weeks).

### 3.2 Counter-Currents (Barriers)

#### 3.2.1 Unsuitable Physical Conditions

One of the points most frequently repeated by the mothers was discomforts of pregnancy and their direct effects on the spiritual care of the unborn child:

3.2.1.1 The Effects of Pregnancy Problems on Reducing Spiritual Behaviors

Pregnancy problems such as fatigue, lethargy, heaviness, having a big abdomen, reluctance to consume food, severe morning sickness, weight loss and edema, particularly in complicated pregnancies such as twin pregnancy will result in the inability to plan precisely for spiritual behaviors or the lack of attention to the unborn child during performing them:

*“I remember when I was pregnant with my son, I had very bad morning sicknesses, I got very weak and my edema was so severe. Perhaps these made me not focus enough on my baby spiritual needs”* (Participant 8, 40 years old, history of two pregnancies).

3.2.1.2 Difficulty in Performing Some Spiritual Considerations

In some cases, complying with spiritual advice was hardly possible for mothers, some suggested foods were not available or it took rather a long time to do some rituals, therefore, due to pregnancy specific conditions, such advice created some problems for mothers:

*“In the information I obtained from books or the Internet, it was advised to read some Quran surahs during pregnancy such as Ad-Dukhānon Fridays, but that was a long surah; or eating a fruit was suggested that was out of season at that time. So I couldn’t use those suggestions”* (Participant 9, 28 years old, history of two pregnancies.

#### 3.2.2 Poor Social and Economic Conditions

Most participants shared that social requirements significantly interfere with spiritual activities of daily living; some of them emphasized on financial problems facing their families:

3.2.2.1 The Negative Effects of Daily Preoccupations

Academic preoccupations, lack of time, occupational conflicts in addition to life management (taking care of husband and children), being away from the family and lack of access to their support as well as family disputes resulted in the reduction of spiritual attention to the unborn child:

*“During my pregnancy, I was studying; I was too busy; I didn’t have a worry-free pregnancy. I was also working. Sometimes I was so tired that after doing daily prayers, I actually passed out, but if I had free time, I’d be more careful in my prayer* (Participant 21, 47 years old, history of one pregnancy).

3.2.2.2 The Negative Effects of Economic Problems

The effects of economic problems are significant in mothers’ and their spouses’ insufficient attention to spiritual behaviors:

*“Now, living conditions are harder than in my previous pregnancy, now everything has become expensive (laughing), it’s a very important factor, my preoccupations have increased and I don’t have time for praying. It’d be good if I did my regular prayers on time”* (Participant 10, 34 years old, second pregnancy, 19 weeks).

#### 3.2.3 Unsuitable Psychological and Cognitive Conditions

Psychological and cognitive problems were concerns expressed by some participants:

3.2.3.1 Lack of Information

Mothers with little information due to studying in non-medical disciplines such as engineering faced with some ambiguities in understanding pregnancy situation, intrauterine environment, the foetus position and his reactions. The lack of easily accessible information resources and lack of adequate information troubled mothers:

*“I had little access to information; I didn’t know many of the things affecting the child’s soul; I always say I wish it was now and I’d observe these things”* (Participant 22, 43 years old, history of one pregnancy).

3.2.3.2 Unintended Pregnancies

Unintended pregnancies resulted in adverse mental conditions and the late start of prenatal care and among them spiritual behaviors:

*“I was more ready for my other children because I intended to have a child. I fasted and read the Quran before my pregnancy, didn’t go everywhere, didn’t eat everything, but this time my pregnancy was out of my control, I was very upset, very (with emphasis), I visited one or two psychiatrists, tried to manage myself a little bit, almost in the fifth months after amniocentesis that I realize the baby would survive, I just started”* (Participant 14, 40 years old, fourth pregnancies, 36 weeks).

#### 3.2.4 Understanding Unsuitable I deological Conditions of the Self and Caregiver

Unsuitable ideological conditions of self were observed in few mothers, but the majority of them were concerned about undesirable ideological conditions in health providers.

3.2.4.1 Ignoring the Effect of Behavioral Changes During Pregnancy on the Unborn Child

Some mothers believed that beliefs and common underlying moral traits are transmitted to the child and behavioral changes during pregnancy have little effect:

*“Whatever is the nature of parents, it will ultimately affect the child, that’s why I didn’t especially change my behavior during pregnancy. I was just trying to keep my spirit up”* (Participant 12, 28 years old, history of one pregnancy).

3.2.4.2 Concerns about the Lack of Caring Physician Interest Toward Spiritual Considerations to the Unborn Child

In some cases, mothers liked to ask their physician to pay spiritual attention to their child such as making ablution before cesarean. But they were extremely concerned that such demands will be rejected by physician or upset them. They did not feel comfortable in asking their spiritual needs and got stressed:

*“I want to ask my doctor to make ablution before my cesarean or do such and such. But I can’t. I’m struggling with myself. I’m afraid she won’t accept or get upset”* (Participant 14, 40 years old, fourth pregnancies, 36 weeks).

3.2.4.3 Understanding Unborn Child Different Needs for Spiritual Considerations during Different Periods of Gestation

Belief in child spiritual need in certain times of pregnancy such as in the late period of pregnancy (because of feeling unborn child movements in response to mother’s behavior) or after the timing of ensoulment (In Islam as the main religion in Iran, ensoulment believed to be at 120 days) led to inadequate attention to the child during whole pregnancy:

*“It’s more effective since the fourth months when the soul enters the child’s body. At that time I was more aware of my behaviors. Before that, he is like a piece of meat and then when he is physically perfect like a man for example in the 6th to 7th months onwards”* (Participant 22, 43 years old, history of one pregnancy).

## 4. Discussion

The findings of this research include eight major categories: “active and passive acquisition of information”, “receiving effective support from people around”, “modeling of self and significant others”, “inner inspirational messages”, “unsuitable physical conditions”, “poor social and economic conditions”, “unsuitable psychological and cognitive conditions”, and finally “understanding unsuitable ideological conditions of the self and caregiver” that, interacting with each other, affect the process of unborn child spiritual care by the mother.

The results showed that the influence of religious teachings, society and media on spiritual self-care behaviors is significant, but the mother herself will attempt voluntarily to obtain information and thus perform “active and passive acquisition of information” in order to provide more comprehensive care to the unborn child. Szwajcer et al. (2005) and Szwajcer et al. (2008) showed that mothers use the Internet, books, midwives and experienced friends to obtain feeding information for protecting their unborn child health. The study of Clarke and Gross (2004) also suggests that in addition to reading books and magazines, recommendations from family and friends are effective in physical activities during pregnancy. Many women from different cultures use the Internet as a primary source of getting information about nutrition, growth and development of the unborn child in order to face with uncertainties related to the pregnancy, prenatal period tests and direct major decisions during pregnancy (Song et al., 2012; Mercer, 1986; Gao et al., 2013). Although exact statistics is not available about interesting information sources for mothers, the number of Internet users during pregnancy and the focus of their search, Iranian women’s cultural and religious context in the present study showed that searching information in addition to physical and medical aspects of pregnancy and birth, encompasses unborn child spiritual development, as mother’s Islamic religious context will provide them basic information in this regard.

“Receiving effective support from people around” was an appropriate motivation for the unborn child spiritual care. Consistent with the results of this study, fathers’ experiences in the study of Sjöling, Hildingsson (2011) showed that the majority of them supported mothers well during pregnancy. Higher social support in Mexican-descent women also led to improved nutrition quality, increased likelihood of using vitamins in the prenatal period and decreased smoking during pregnancy (Harley & Eskenazi, 2006).

Mother’s experience suggests that “modeling of self and significant others” on the formation of her spiritual beliefs and behaviors is very influential. In this regard, Evans (2001) also showed that Australian pregnant teens follow their mothers’ and sisters’ decision-making models to continue pregnancy or abort the unborn child.

Unborn child responses in the form of specific movements to mother’s behaviors, feeling the child presence and monitoring and mother’s inner responsibility towards the child future create inner messages that refresh mother and make her motivation and performance double to adopt spiritual behaviors. Studies have also shown that the higher maternal-fetal attachment is associated with decreased high risk behaviors such as smoking and drug use in pregnancy (Alhusen, 2008; Magee et al., 2013, 2002). Since culture is an important variable that can affect the process of maternal-fetal relationship and maternal role development (Mercer, 1986), examining its effect on maternal behaviors in different cultural, social and religious contexts will provide useful information for improving mother’s health and the child future.

Unsuitable physical, economic, social, psychological, cognitive and ideological conditions were identified barriers of the unborn child spiritual care by the mother.

Personal, economic-social, organizational and cultural barriers such as the lack of information, child care problems, work commitments, having not enough money or insurance for prenatal visits and not being able to make an appointment when they wanted, lack of monthly income, young age, a large number of children, foreign nationality, not being married, unplanned pregnancies, low education level, having difficulty in dealing with health services organizations and care of children were reported as the focus of health care providers on problematic aspects of high-risk pregnancies rather than the positive aspects of pregnancy and birth as barriers of prenatal care during pregnancy of European and American mothers (O’Higgins et al., 2014; Kitsantas et al., 2012; Delvaux et al., 2001).

Review study of Phillippi (2009) on American women perception of barriers in prenatal care from 1999 until the time of study also showed maternal and social barriers such as the lack of enough motivation for caring in unintended pregnancies, fear of medical procedures or disclosing pregnancy for others, depression or lack of belief in the necessity for caring and structural barriers including long waiting time, clinic location and working hours, clinic staff’s language and attitudes and service providers, service prices and shortage of child-friendly facilities. In some cases, mothers take responsibility for self-care behaviors during pregnancy for keeping their health; activities such as walking, jogging, changing food diet, working, exercising and meditation (Hawkins et al., 1998; Gollenberg et al., 2008).

The results suggest that Iranian mothers in addition to having similar ideas with other cultures in prenatal care adopt self-care behaviors in the area of the unborn child spiritual care influenced by personal emotions, beliefs and values that is rooted in Iranian religion and culture. Iranian women believe that formation of child’s spiritual and moral education begins from foetal period, so they try to provide a favorable environment for their mental development in addition to paving the way for the child physical growth and development. In Islam, as the dominant religion of Iranian women, this issue has been emphasized significantly (Majlesi, 2005).

The findings of this research explain many facilitators and barriers of the unborn child spiritual care in Iranian pregnant mothers. These findings can inform the midwifery community and other health professionals of different factors affecting unborn child spiritual care by mother so that they can benefit from them in assessment and holistic prenatal care based on pregnant mothers’ worldview. So, in prenatal services, it is better to encourage appropriate training in the form of prenatal courses, using social media and enrichment of Internet resources, providing supportive solutions such as facilitating family, peer and community support, reassuring mothers of their ability to perform motherhood responsibilities for caring the child as much as possible to facilitate the involvement and participation of mothers in comprehensive prenatal care.

Using interdisciplinary professionals’ skills such as clinical psychologists, sociologists, clergymen, researchers, policy makers and planners of providing maternal and child health services in order to overcome psychological, cultural, economic, social barriers proportional to pregnant women’s cultural, religious and social sensitivities can result in providing holistic prenatal care to improve self-care behaviors, mother and the unborn child physical and spiritual empowerment and creating an human desired future.

Although data from qualitative studies are strongly influenced by participants’ religious, cultural and social context, we tried to help transferability of results through creating the highest possible diversity in the participants.

## 5. Conclusion

Iranian cultural and religious perspective on the unborn child physical and spiritual influence from mother has an important role in mother’s self-care behaviors during pregnancy. It seems that understanding facilitators and barriers of mother’s care of the unborn child can help designing an interdisciplinary program based on training and support and can lead to providing comprehensive prenatal care according to mothers’ cultural, religious and social context and promoting their capabilities to holistic care their unborn child.
